# Establishment of 2.5D organoid culture model using 3D bladder cancer organoid culture

**DOI:** 10.1038/s41598-020-66229-w

**Published:** 2020-06-10

**Authors:** Amira Abugomaa, Mohamed Elbadawy, Megumi Yamanaka, Yuta Goto, Kimika Hayashi, Takashi Mori, Tsuyoshi Uchide, Daigo Azakami, Ryuji Fukushima, Toshinori Yoshida, Makoto Shibutani, Risako Yamashita, Mio Kobayashi, Hideyuki Yamawaki, Yuta Shinohara, Masahiro Kaneda, Tatsuya Usui, Kazuaki Sasaki

**Affiliations:** 1 Laboratory of Veterinary Pharmacology, Department of Veterinary Medicine, Faculty of Agriculture, Tokyo University of Agriculture and Technology, 3-5-8 Saiwai-cho, Fuchu, Tokyo 183-8509 Japan; 2 Faculty of Veterinary Medicine, Mansoura University, 35516 Mansoura, Egypt; 3 Department of Pharmacology, Faculty of Veterinary Medicine, Benha University, 13736 Moshtohor, Toukh Egypt; 4 Laboratory of Veterinary Clinical Oncology, Faculty of Applied Biological Sciences, Gifu University, 1-1, Yanagido, Gifu, Gifu, 501-1193 Japan; 5 Center for Highly Advanced Integration of Nano and Life Sciences, Gifu University (G-CHAIN), 1-1, Yanagido, Gifu, Gifu, 501-1193 Japan; 6 Department of Veterinary Surgery, Faculty of Agriculture, Tokyo University of Agriculture and Technology, 3-5-8 Saiwai-cho, Fuchu, Tokyo 183-8509 Japan; 7 Department of Veterinary Clinical Oncology, Faculty of Agriculture, Tokyo University of Agriculture and Technology, 3-5-8 Saiwai-cho, Fuchu, Tokyo 183-8509 Japan; 8 Animal Medical Center, Faculty of Agriculture, Tokyo University of Agriculture and Technology, 3-5-8 Saiwai-cho, Fuchu, Tokyo 183-8509 Japan; 9 Laboratory of Veterinary Pathology, Department of Veterinary Medicine, Faculty of Agriculture, Tokyo University of Agriculture and Technology, 3-5-8 Saiwai-cho, Fuchu, Tokyo 183-8509 Japan; 10 Laboratory of Veterinary Pharmacology, School of Veterinary Medicine, Kitasato University, 35-1 Higashi 23 ban-cho, Towada, Aomori 034-8628 Japan; 11 Pet Health & Food Division, Iskara Industry CO., LTD, 1-14-2, Nihonbashi, Chuo-ku, Tokyo 103-0027 Japan; 12 Laboratory of Veterinary Anatomy, Department of Veterinary Medicine, Faculty of Agriculture, Tokyo University of Agriculture and Technology, 3-5-8 Saiwai-cho, Fuchu, Tokyo 183-8509 Japan

**Keywords:** Cancer, Stem cells, Oncology, Urology

## Abstract

Three-dimensional (3D) organoid culture holds great promises in cancer precision medicine. However, Matrigel and stem cell-stimulating supplements are necessary for culturing 3D organoid cells. It costs a lot of money and consumes more time and effort compared with 2D cultured cells. Therefore, the establishment of cheaper and Matrigel-free organoid culture that can maintain the characteristics of a part of 3D organoids is demanded. In the previous study, we established a dog bladder cancer (BC) 3D organoid culture system by using their urine samples. Here, we successfully isolated cells named “2.5D organoid” from multiple strains of dog BC 3D organoids using 2.5 organoid media. The cell proliferation speed of 2.5D organoids was faster than parental 3D organoid cells. The expression pattern of stem cell markers was close to 3D organoids. Injection of 2.5D organoid cells into immunodeficient mice formed tumors and showed the histopathological characteristics of urothelial carcinoma similar to the injection of dog BC 3D organoids. The 2.5D organoids had a similar sensitivity profile for anti-cancer drug treatment to their parental 3D organoids. These data suggest that our established 2.5D organoid culture method might become a reasonable and useful tool instead of 3D organoids in dog BC research and therapy.

## Introduction

Bladder cancer (BC), also known as transitional cell carcinoma (TCC) or urothelial carcinoma, is the most frequent form of urinary BC in dogs, affecting thousands of dogs annually worldwide^1^. It constitutes approximately 1–2% of all naturally occurring cancers in dogs, a nearly similar rate to humans^2,3^. The majority of dog BC is high grade invasive urothelial carcinoma^2,4,5^. In addition to the late diagnosis of dog BC, it is very difficult to treat them^6,7^. Although several experimental models of BC by using mouse and rat were developed^8–10^, they hardly reflect the characteristics of invasive or metastatic human BC^11^ and the mechanism of its arising and development still remains obscure. Interestingly, the naturally arising dog BC closely mimics invasive BC in human in the cellular and molecular features like histopathological architecture, local invasion, distant metastases, chemotherapy response, and prognosis^7^, suggesting that dog BC might be a pertinent model of invasive BC in human.

Three-dimensional (3D) organoid culture is derived from self-renewing stem cells that typically recapitulate the *in vivo* architecture, functions, and genetic and molecular imprints of their original tissues. It holds great promise for use in medical research for establishing novel personalized therapies, especially cancer^12,13^.

In the previous study, we established the 3D culture method of dog BC organoids using the urine samples from BC diseased dogs^14^. The organoids recapitulated the tumor microenvironment of their parental tumor tissues and showed tumorigenesis *in vivo*. Furthermore, it could be used to investigate the sensitivity of anti-cancer drugs and their combinations. However, using the 3D organoid culture method for experiments costs a lot of money due to the expensive gel and the stem cell-stimulating supplements contained in the culture media. It also needs a long time because of the complicated handling (dissolving and solidifying gel) and the slow growth speed of the organoid cells compared with 2D cell lines. Although there is a need to establish a cheaper and easier-to-use organoid culture method, an alternative to the 3D organoid culture method has not been fully developed.

In the present study, we hypothesized that some media components enable patient-derived 3D organoid cells to culture in Matrigel-free 2D culture conditions without losing their characteristics such as marker expression, stemness, and drug sensitivity of their original 3D organoids (we named the cells 2.5D organoids). Here, we identified the suitable media components for culturing 2.5D organoids and established a new culture method of dog BC 2.5D organoids from different strains of dog BC 3D organoids. Since these 2.5D organoids partially exhibited similar characteristics to their parental 3D organoids, we expect that they could be useful as a new experimental tool not only in dogs but also in human BC research.

## Materials and Methods

### Materials

To isolate 2.5D organoids from 3D organoids, we used a special 2.5D organoid media. The components were as follows: Advanced Dulbecco’s Modified Eagle’s Medium (DMEM)/F12 (Thermo Fisher Scientific Inc., Waltham, MA, USA) supplemented with 10 mM 4-(2-hydroxyethyl)-1-piperazineethanesulfonic acid (HEPES;WAKO, Osaka, Japan), 1% GlutaMax (Thermo Fisher Scientific Inc), 10 mM Nicotinamide (Sigma-Aldrich, St. Louis, MO, USA), 1 mM N-acetyl-L-cysteine (Sigma-Aldrich), 0.5 μM A83-01 (Adooq Bioscience, Irvine, CA, USA), 1% penicillin-streptomycin (PS; WAKO), and 5% fetal bovine serum (FBS; Thermo Fisher Scientific Inc.). The difference of culture supplements between 2.5D and 3D organoid was shown in Table 1. Anti-cancer drugs were as follows: vinblastine, mitoxantrone (Cayman, Ann Arbor, MI, USA), and carboplatin (WAKO). The used antibodies were as follows: CK5 (GeneTex, Inc., Irvine, CA, USA), CK7 (Novus, Centennial, CO, USA); CK20 and UPK3A (Bioss, Woburn, MA, USA). Fluorescent secondary antibodies were as follows: Alexa Fluor 488 goat anti-rabbit IgG; Alexa Fluor 488 goat anti-mouse IgG; (Thermo Fisher Scientific Inc.). Dog urothelial carcinoma cell lines were purchased from COSMO BIO CO., Ltd (Tokyo, Japan), and cultured in DMEM containing 10% FBS (Thermo Fisher Scientific Inc.) and 1% Penicillin-Streptomycin (PS) (WAKO).

**Table 1:** The difference of media components between 2.5D and 3D organoid culture.

Supplement	2.5D organoid	3D organoid
Wnt, Noggin and R-Spondin	−	+
FBS	+	−
EGF	+	+
GlutaMax	+	+
N-Acetyl-l-cysteine	+	+
Nicotinamide	+	+
A83-01	+	+
HEPES	+	+
Antibiotics	+	+

### Generation of dog BC 2.5D organoids

In the previous study, we generated urine sample-derived dog BC 3D organoids^14^. Using our established 3 strains of BC organoids (Table 2), we generated 2.5D organoids in the present study. Each dog 3D organoid cell was treated with 2.5D organoid media and cultured for 3–7 days. After the migrated cells from organoids attached the bottom of the plate, Matrigel containing organoids was stripped and 500 µL of 5 mM ethylenediaminetetraacetic acid (EDTA)/ Phosphate Buffered Saline (PBS) was added per well for 10 min at 37 °C in a CO_2_ incubator. The EDTA solution in each well was collected in a 15 ml tube and centrifuged at 600 *g* for 5 min. After the cell pellet was trypsinized for 3 min, they were seeded in 6 cm dishes with our prepared 2.5D organoid media. Media were changed three times a week. The present study was conducted under the institutional Animal Care and Use Committee of Tokyo University of Agriculture and Technology approval (Approval number: 0016012).

**Table 2:** Sample information.

Case ID	Age (year old)	Breed	Sex	Sample Date
BC18004 (used as BC1)	12	Miniature Dachshund	Female (spayed)	2018/5/2
BC18005 (used as BC2)	11	Mix	Female (spayed)	2018/5/11
BC19004 (used as BC3)	11	Maltese	Female (not spayed)	2019/8/2

### Passaging of dog BC 2.5D organoids

After 70–80% confluent conditions, 2.5D organoid cells were passaged into a new 6 cm dish at a ratio of 1:3–4. To collect cells, 1 mL of 5 mM EDTA/PBS was added, and the culture dish was placed at CO_2_ incubator at 37 °C for 10 min. Thereafter, the EDTA solution was collected. Subsequently, 1 mL TrypLE Express solution (Thermo Fisher Scientific Inc.) was added to the cell pellets and they were incubated at 37 °C for 3–5 min. The trypsinized cell suspension was vigorously pipetted and collected into a 15 mL tube containing 100 μl FBS and then centrifuged at 600 *g* for 3 min. Cell pellets were mixed with new 2.5D organoid media and seeded into a 6 cm dish. We defined the passaged cells as early (4–8) and late passage (15–20) cells.

### Comparison of culture efficiency between 2.5D organoid media and 2D cell line media

After the isolated 2.5D organoid cells were seeded into 6 cm dishes, they were cultured with 2.5D organoid media or 2D cell line media (DMEM containing 10% FBS and 1% PS) to compare the efficiency of cell attachment and proliferation after passaging. The bright-field images were obtained using a light microscope (BX-52; Olympus, Tokyo, Japan).

### Immunofluorescence staining of 2.5D organoids

Immunofluorescence staining of BC 2.5D organoids was performed as described before^14,15^. Briefly, the 2.5D cells (2 × 10^5^/well) were seeded on a coverslip in a 6-well plate. After 70% confluent conditions, the cells were fixed with 4% paraformaldehyde (PFA) solution for 10 min. Later, the cells were exposed to 0.2% Triton X/PBS for a few seconds. Blocking was done using 3% skimmed milk/PBS for 1 h. Subsequently, cells were treated with the primary antibodies (CK5; 1:200, CK7;1:50, CK20;1:200, and UPK3A;1:200) and kept at 4 °C overnight. After they were treated with the secondary antibodies and DAPI solution (1:1000) for 1 h at room temperature, the expressions were examined using a confocal microscope (LSM 800; ZEISS, Copenhagen, Germany).

### Cell proliferation assay

After an equal number of 2.5D organoid cells at early and late passages and 3D organoid cells were seeded in 96-well plate at a density of 2×10^3^ cells/well, each cell was cultured for 5 days. The number of living cells at day 1, 3 and 5 was evaluated by using a Prestoblue kit (Thermo Fisher Scientific Inc.). The fluorescence intensity was measured by a microplate reader (TECAN, Seestrasse, Switzerland) at an emission wavelength of 585 nm.

### Quantitative real-time PCR

Quantitative real-time PCR was performed as described previously^14^. Total RNA was extracted from 2 × 10^5^ of 2.5D organoid cells at early and late passage, 3D organoids, and urothelial carcinoma cell lines by using a NucleoSpin kit (Takara Bio Inc., Shiga, Japan) following the manufacturer’s protocol. First-strand cDNA was prepared using a QuantiTect Reverse Transcription Kit (TOYOBO, Tokyo, Japan). Quantitative real-time PCR was done using a QuantiTect SYBR I Kit (QIAGEN, Hilden, Germany) and a StepOnePlus Real-Time PCR System (Applied Biosystems, Waltham, MA, USA). The ΔΔCq method was used for quantification. Specific primers used for dog BC stem cell markers, SOX2, CD44, and GAPDH were designed and are shown in Table 3.

**Table 3:** Primers for real-time quantitative PCR analysis.

Gene name	Primer	Sequence
SOX2	Forward	5′-GCCCTGCAGTACAACTCCAT-3′
	Reverse	5′-GGAGTGGGAGGAGGAGGTAA-3′
CD44	Forward	5′-CCAAGACAGTTCCAGGGTGT-3′
	Reverse	5′-TTGAGGTTTCCGCATAGGAC-3′
GAPDH	Forward	5′-AACTCCCTCAAGATTGTCAGCAA-3′
	Reverse	5′-CATGGATGACTTTGGCTAGAGGA-3′

### Mouse xenograft assay

Mouse xenograft assay of 2.5D organoids was carried out as previously described^14,15^. Four NSG male immunodeficient mice (6 weeks old) were obtained from Japan SLC (Shizuoka, Japan). They were housed under strict pathogen-free conditions. Here, 1×10^6^ 2.5D organoid cells were subcutaneously injected into the back of mice. Six weeks later, the 2.5D organoid-derived tumors were dissected under isoflurane anesthesia and used for H&E and immunofluorescence staining. All animal experiments in the present study were performed following the Guide to Animal Use and Care of Tokyo University of Agriculture and Technology and approved by the ethics committee (Approval number: 29–92).

### H&E staining of xenografted tumor tissues

H&E staining of 2.5D organoid-derived tumor tissues was performed as described previously^14,15^. After the isolated tumor tissues were fixed with 4% PFA for 24 h, they were embedded in paraffin. After deparaffinization, 4 µm-thick sections were stained with H&E. The images were obtained using a light microscope (BX-52; Olympus).

### Sensitivity of 2.5D organoids to anti-cancer drugs

Cell viability assay of the 2.5D organoid cells at early and late passages compared with their original 3D organoid ones was carried out as described before^14^. Briefly, 1 × 10^3^ cells of each 2.5D cells or 2 × 10^3^ of their 3D organoid single cells were seeded in 96-well culture plates and incubated in a CO_2_ incubator at 37 °C for 24 h. The cells were treated with anti-cancer drugs including a microtubule inhibitor, vinblastine (0.01–10 nM), a topoisomerase inhibitor, mitoxantrone (0.1–100 ng/mL), or a DNA-damaging agent, carboplatin (0.1–100 µg/mL) for 72 h. Cell viabilities of the 3D and 2.5D organoid cells were examined in the same way as in the cell proliferation assay.

### Statistical analysis

The obtained data were presented as means ± SEM. Statistical assessments were carried out using the one-way analysis of variance (ANOVA) method followed by Bonferroni’s test. Statistical significance was considered when *P*-values ≤ 0.05.

## Results

### Generation of dog bladder cancer (BC) 2.5D organoids

**Figure 1:**
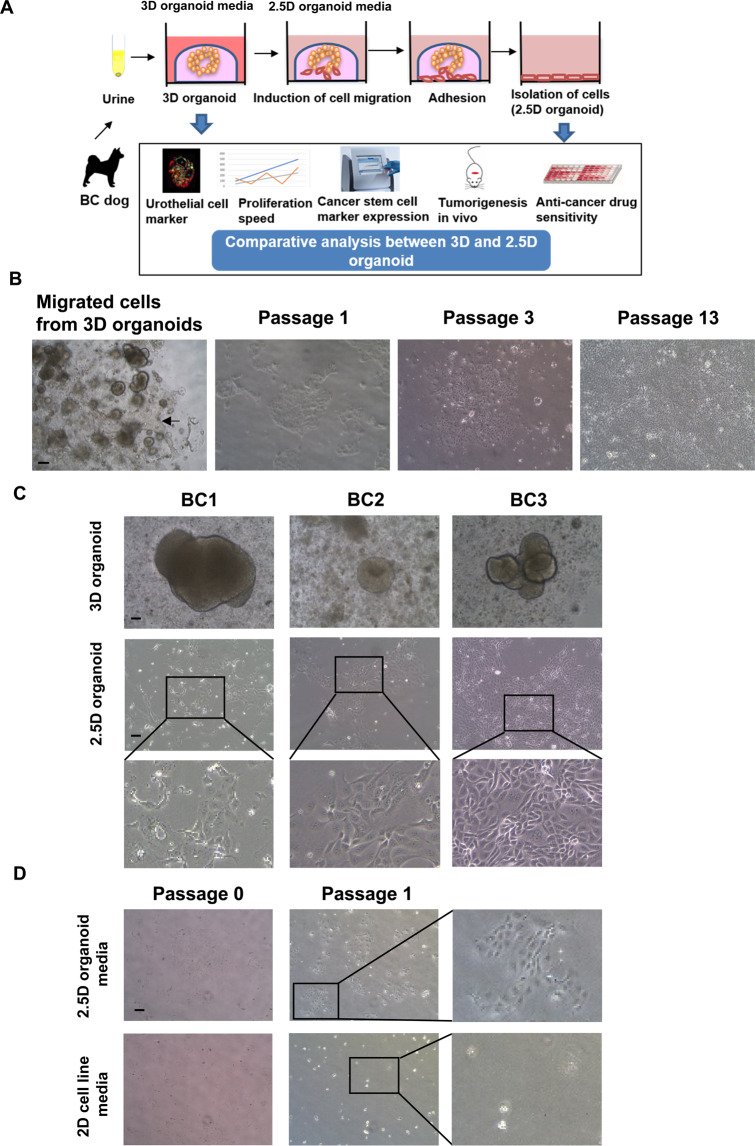
Generation of dog bladder cancer (BC) 2.5D organoids. Schematic experimental design of a procedure for isolation of 2.5D organoid cells from 3D organoids and the analysis overview (**A**). Representative bright-field images of the process of generation of 2.5D organoids from 3D ones and serially passaged cells (**B**). Scale bar: 500 μm. Representative images and enlarged ones (Scale bar: 200 μm) for three different strains of 2.5D organoid cells and their parental 3D organoids (**C**). Comparison of cell attachment and proliferation between the 2.5D organoid media and 2D cell line media (**D**). Representative images at passage 0 and 1 and enlarged ones at passage 1 were shown. Scale bar: 500 μm.

In the previous study, we established a novel method of 3D organoid culture from BC diseased dogs using their urine samples^14^. Our established BC 3D organoids expressed urothelial markers (CK7, CK20, and UPK3A), showed the response to anti-cancer drugs, and exhibited tumorigenesis. In the present study, we hypothesized that some media components enable 3D organoid cells to culture in gel-free 2D culture conditions without losing their characteristics such as marker expression, stemness, and drug sensitivity. To prove the hypothesis, we made original media and checked whether the media efficiently generates 3D organoid-derived 2D cells (2.5D organoid) and can keep the characteristics (Fig. 1A). After treating 3D organoid cells with the media for a few days, cells gradually migrated from organoids and adhered to the bottom of the plate (Fig. 1B). We purely isolated the adhered cells and cultured them in 2.5D organoid media. The 2.5D organoid cells were successfully passaged and propagated over passage 10 (Fig. 1B). We also confirmed that 2.5D organoids can be isolated from different strains of dog BC 3D organoids (Fig. 1C). The cells have a cobblestone- and fibroblast-like appearance (Fig. 1C), similarly to urothelial carcinoma cell lines^16^. To prove the utility of 2.5D organoid media, we compared the cell attachment and growth between 2.5D organoid and normal cell line media (Fig. 1D). The isolated 2.5D organoid cells cultured in 2.5D organoid media showed a better cell attachment and proliferation than normal cell line media (Fig. 1D). Further, we tried to generate 2.5D organoids directly from urine samples without going through 3D organoids. However, the cells did not proliferate well (data not shown), suggesting that the generation of 3D organoids at first is a necessary step for generating 2.5D ones.

### Expression of urothelial markers in BC 2.5D organoids

**Figure 2:**
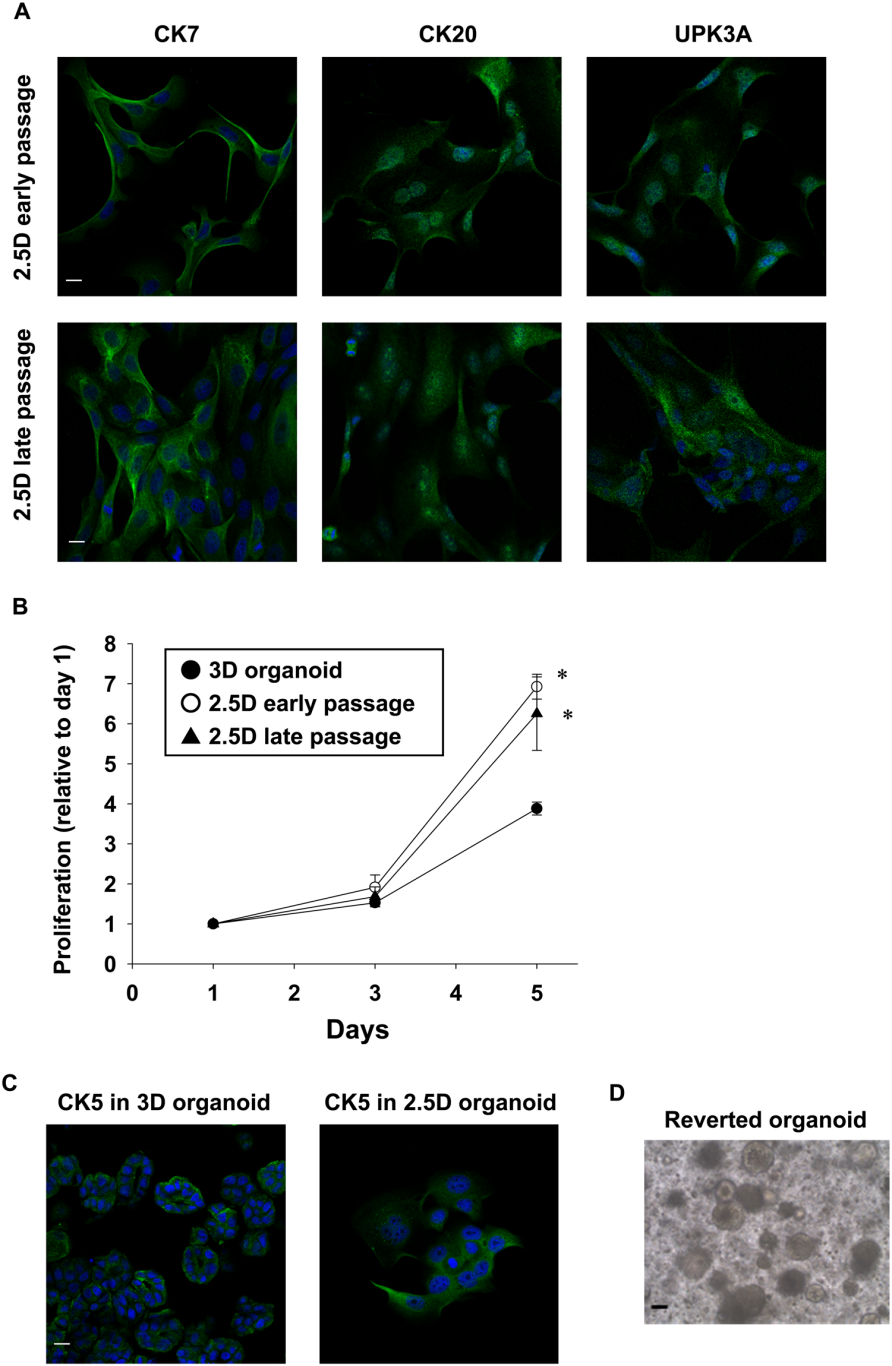
Characterization of BC 2.5D organoids. Expression of urothelial cell markers, CK7, CK20, and UPK3A in 2.5D organoid cells at early (4–8) and late passages (15–20) (**A**, n = 4). Scale bar: 50 μm. Comparison of cell proliferation at day 1, 3, and 5 between 2.5D organoid at the early and late passage and their original 3D organoid. Cell proliferation was assayed by Prestoblue cell viability reagent and shown as fold increase relative to day 1 (**B**, n = 6). Results were expressed as mean ± S.E.M. *P < 0.05 vs. 3D organoid. Expression of a basal cell marker, CK5 in the 2.5D organoid cells and parental 3D organoids (**C**, n = 4). Scale bar: 50 μm. Plasticity of BC 2.5D organoids. Representative bright-field images of the reverted 3D organoids from 2.5D ones (**D**). Scale bar: 200 μm.

To identify the characterization of BC 2.5D organoid cells, we investigated the expression of urothelial markers (CK7, CK20, and UPK3A). The 2.5D organoid cells (both early and late passages) expressed urothelial cell markers, CK7, CK20, and UPK3A (Fig. 2A) similar to the original BC 3D organoids^14^.

### Cell proliferation speed in BC 2.5D organoids

After seeding equal numbers of 2.5D organoid cells at early and late passages as well as their original 3D organoids, living cell number was evaluated by Prestoblue assay. The 2.5D organoid cells at early and late passages showed a significantly accelerated proliferation rate at day 5 compared with their original 3D organoids (Fig. 2B).

### Expression of basal cell marker in BC 2.5D organoids

To check whether a basal cell marker was expressed in 2.5D organoids, we investigated the expression of a basal marker, CK5. The 2.5D organoid cells expressed CK5 similarly to the parental 3D organoids (Fig. 2C).

### Plasticity of BC 2.5D organoids

To check whether 2.5D organoids can revert to 3D organoids, we collected the generated 2.5D organoid cells and embedded them into Matrigel again. The 2.5D organoid cells were successfully reverted to 3D organoids (Fig. 2D), which were continually expanded after passaging, indicating that 2.5D organoid cells maintain the stemness property and possess the plasticity.

### Expression pattern of cancer stem cell markers, SOX2 and CD44 in BC 2.5D organoids

**Figure 3:**
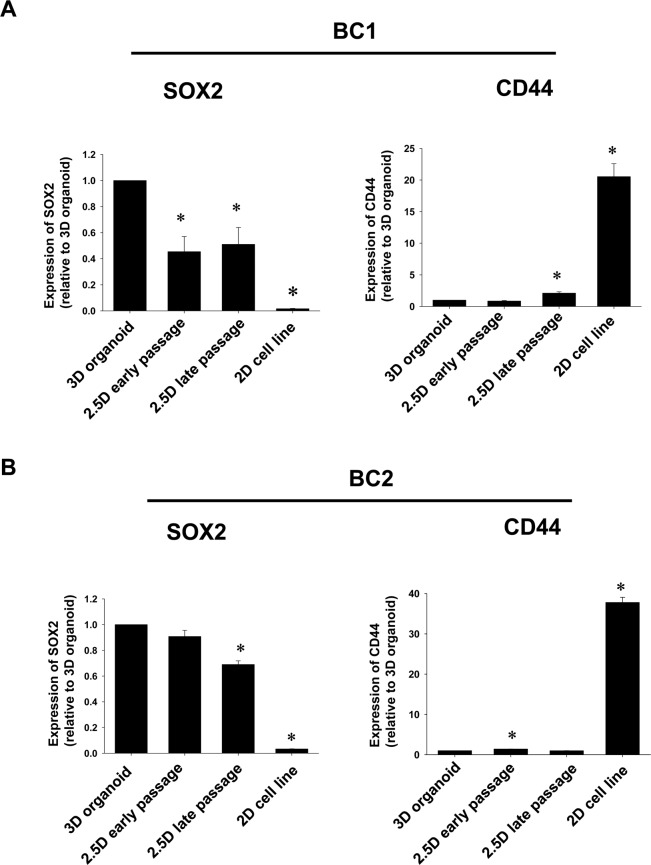
Comparison of cancer stem cell markers, SOX2 and CD44 between BC 2.5D organoid at the early and late passage and their original BC 3D organoids. Dog urothelial carcinoma cells were used as 2D cell lines. Expression level of SOX2 and CD44 in 3D and 2.5D organoids from different strains (BC1; **A** and BC2; **B**) and 2D cell lines was analyzed by quantitative real-time PCR (n = 4) and quantified based on the ratio of expression level to GAPDH. Data were expressed as mean ± SEM. *P < 0.05 vs. 3D organoid.

Since 3D organoids contain many stem cells compared with 2D cultured cells, we investigated whether BC 2.5D organoids maintain the expression level of stem cell markers as an indicator of stemness. Among 3D organoid, 2.5D early passage organoid, 2.5D late passage organoid, and urothelial carcinoma cell line, mRNA expression level of SOX2 was the highest in 3D organoid (Fig. 3A). In both 2.5D early passage and 2.5D late passage organoids, mRNA expression of SOX2 was a little bit lower compared with 3D organoids but much higher than cell lines (Fig. 3A). Interestingly, mRNA expression level of CD44 was the highest in cell line. In both early and late passages of BC 2.5D organoids, expression level of CD44 was quite lower similar to the parental 3D organoid (Fig. 3A). In another strain of BC organoid, we confirmed that stem cell marker expression showed the similar trend (Fig. 3B). These results suggest that 2.5D organoids could keep the characteristic of stemness in 3D organoids.

### Tumorigenesis induced by BC 2.5D organoids

**Figure 4:**
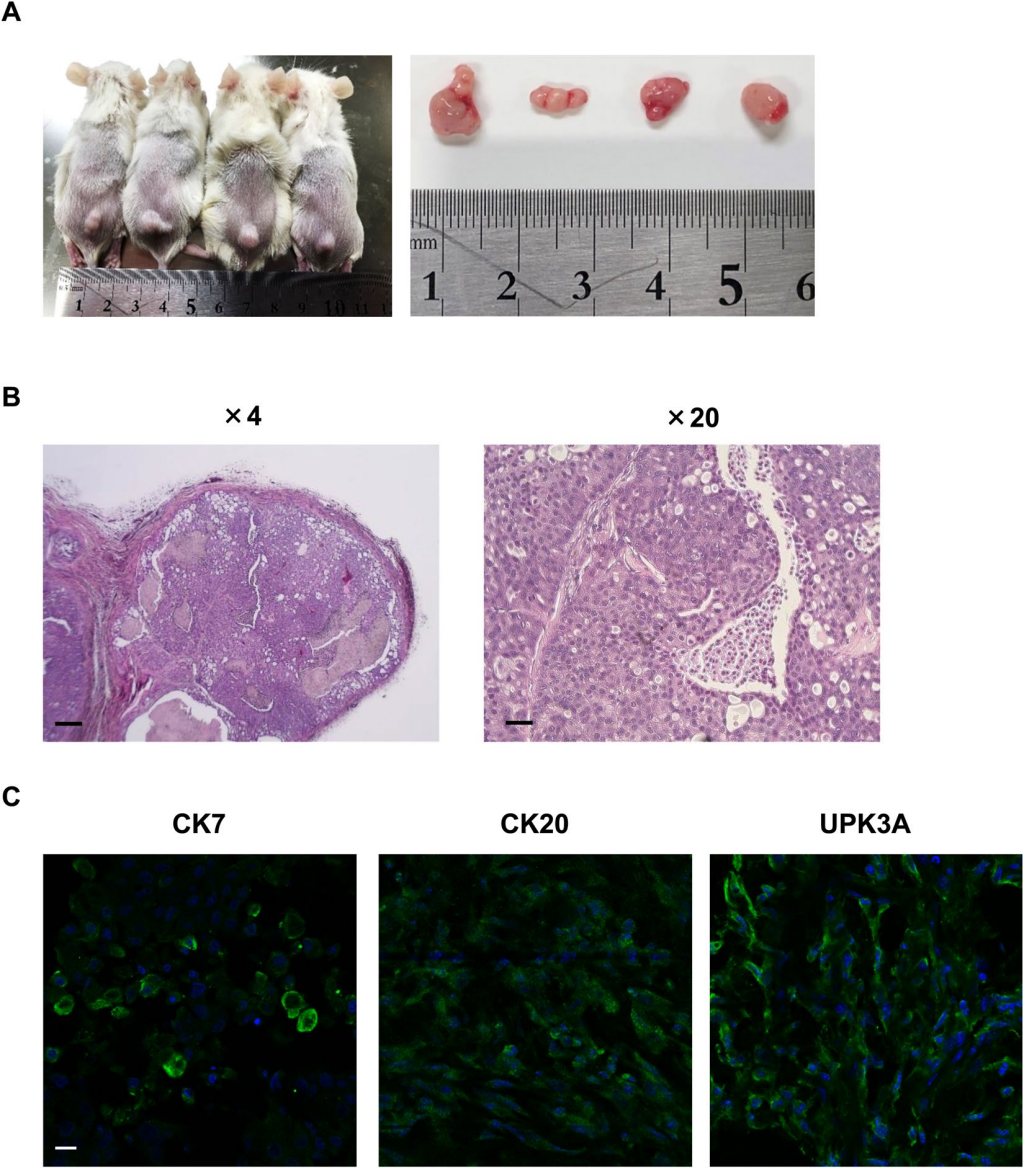
Tumorigenesis induced by BC 2.5D organoids. The trypsinized 2.5D cells (1×10^6^) were subcutaneously injected into the back of NSG mice (n = 4). Six weeks later, the formed tumors were isolated and sectioned for H&E and immunofluorescence staining. Representative image of the formed tumors and their sizes (**A**). Representative images of H&E staining of the tumor tissue sections. The enlarged image is shown on the right. Scale bar: 500 and 100 μm (**B**). Characterization of the cellular components of the formed tumors. Expression of CK7, CK20, and UPK3A in the tumor tissues (**C**). Representative photomicrographs were shown. Scale bar: 50 μm.

In the previous study, we demonstrated that urine sample-derived BC organoids formed a tumor *in vivo*^14^. We therefore checked the tumorigenesis of the 2.5D organoids *in vivo*. After subcutaneous injection of the 2.5D cells into the back of immunodeficient mice, tumors of less than 1 cm in diameter were successfully formed after 6 weeks (Fig. 4A). Additionally, H&E staining of the formed tumors showed the histopathology of typical urothelial carcinoma (Fig. 4B). To characterize the cellular components of the isolated tumor tissues, immunofluorescence staining was carried out and expression of CK7, CK20, and UPK3A was observed in the tumor tissues (Fig. 4C). These findings suggest that BC 2.5D organoids maintained the ability to form tumors *in vivo* similar to BC 3D organoids.

### Response to anti-cancer drugs in BC 2.5D organoids

**Figure 5:**
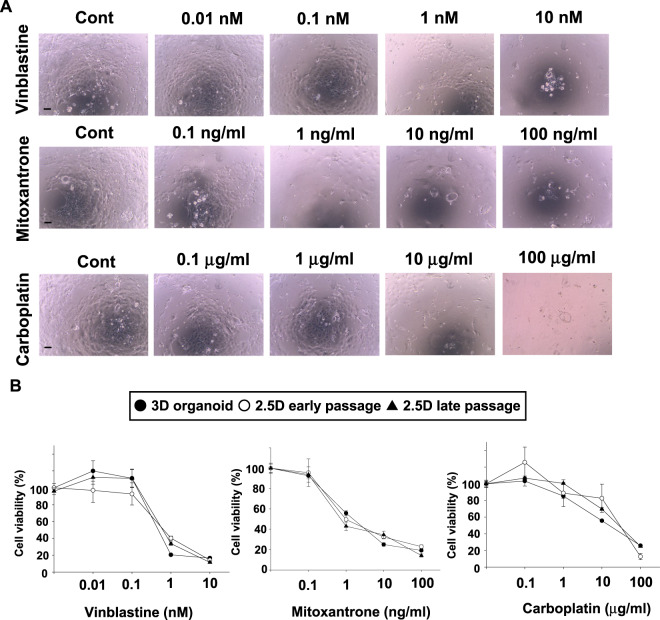
Comparison of the anti-cancer drug sensitivity between BC 2.5D organoids at the early and late passage and their original 3D organoids. After the 2.5D organoids were trypsinized and seeded into 96 well plates, they were treated with vinblastine, mitoxantrone, and carboplatin for 72 h. Representative phase-contrast images of the treated 2.5D organoid cells at early passage were shown (**A**). Scale bar: 500 μm. Cell viability was assayed by the Prestoblue cell viability kit and 100% represents the cell viability of each control (**B**, n = 6). Data were presented as mean ± S.E.M.

Since it was shown that dog BC 3D organoids can apply the anti-cancer drug sensitivity test in our previous study^14^, we compare the response to anti-cancer drugs between 2.5D organoids and their original 3D organoids. After 72 h treatment with different concentrations of a microtubule inhibitor, vinblastine, a topoisomerase inhibitor, mitoxantrone, and a DNA-damaging agent, carboplatin, the cell viability of 2.5D organoid cells at early and late passages decreased in a dose-dependent manner and showed a similar responsive profile to their original 3D organoids (Fig. 5A,B), suggesting that our established 2.5D organoids maintain the anti-cancer drug response profiles of their original 3D organoids and could be used for anti-cancer drug sensitivity test.

## Discussion

In the current study, we for the first time established a new culture method of dog BC 2.5D organoids. These 2.5D organoids at even early and late passage maintained several characteristics of their parental 3D organoids. The main findings of the current study are as follows: (1) 2.5D organoid cells were successfully isolated from the different strains of 3D organoids by using our identified 2.5D organoid media (Fig. 1). (2) 2.5D organoids maintained the expression of urothelial cells markers, CK7, CK20, and UPK3A (Fig. 2A) and their proliferation speed was significantly higher than their parental 3D organoids (Fig. 2B). (3) Expression level of a cancer stem cell marker, SOX2 in 2.5D organoids was lower than 3D organoids but much higher than 2D urothelial carcinoma cell lines (Fig. 3). (4) Injection of 2.5D organoid cells into NSG mice successfully generated tumors (Fig. 4). (5) 2.5D organoids showed a similar response to anti-cancer drugs compared with their parental 3D organoids (Fig. 5). Collectively, our data indicate that dog BC 2.5D organoids could be useful to investigate the mechanisms of dog BC and provide new insights for dog BC therapy.

The 3D organoid culture models have become charming stuff for assaying cancer cell proliferation, differentiation, metabolism, tumor-stroma crosstalk, invasion, metastasis, and drug sensitivity screening^17–21^. The 3D culture system has thus gotten more attention as a tool to overcome the drawbacks of the 2D culture system for predicting the *in vivo* activities and by recapitulating the tumor microenvironment^22^. Although the 3D culture method has had a strong impact on the *in vitro* study of tumor biology and its therapeutic potential, it meets significant challenges. The Matrigel used in organoid culture is derived from animals with undefined hydrogel matrix compositions and has limited broad applications for the 3D organoid culture system in mechanistic and clinic-related studies besides its expensive cost and the long time it takes for seeding and growing the organoids. To overcome some of these limitations, researchers have focused back on 2D monolayer culture systems. Recently, Puca *et al*., named the cells derived from 3D prostate organoids as 2D organoids because the cells maintained the purity and genomic profile of their parental 3D organoids and also the original tumor tissues at serial timepoints with a concordant genome-wide copy number alteration^23^. In addition, in the previous published 2.5D organoid paper^24^, the cells were grown on top of a thick layer of ECM proteins such as Matrigel that allowed for tissue-specific differentiation of a variety of cells. Nevertheless, the culture system could not perfectly model the *in vivo* environment. Despite these limitations, 2.5D organoid assays are experimentally convenient and can induce cells to form a more physiological tissue architecture than conventional 2D cell culture systems. In the present study, we for the first time established a new gel-free culture method of dog BC cells, namely 2.5D organoid culture, that is considered as a phase between 2D and 3D. The cells were originally derived from their parental 3D organoids, cultured in special media that differ from organoid media, and grown rapidly and efficiently for several passages (Fig. 1). Since our established gel-free 2.5D organoid could recapitulate most of the characteristics of their parental 3D organoids, it is clearly different from previous models. Furthermore, our newly established method overcame the issues of high cost and long handling time of organoids. Therefore, we believe that our established method becomes a promising research model for studying patient-derived cancer cells.

The difference of the media components between the 2.5D and 3D BC organoids was presented in Table 1. The presence of 5% FBS and EGF in the 2.5D media promoted cell growth, migration, differentiation, and attachment to the bottom of culture dish^25–27^. Further, a TGF-β inhibitor, A83-01, was added to prevent fibroblast proliferation. Additionally, the 2.5D organoid culture media do not contain the strong stemness-supporting supplements, such as Wnt, Noggin, and R-spondin contained in the 3D organoids culture media. Other supplements such as nicotinamide and N-acetyl-L-cysteine were used to maintain the stemness. We suppose that these components are important for generating 2.5D BC organoid cells.

Characterization of the normal and neoplastic epithelium of the urinary bladder has been performed mainly using cytokeratins (especially CK7 and CK20) and UPK3A antibodies in human^28^ and dogs^29^. They are useful as diagnostic aids in carcinoma of the bladder and their co-existent metastasis^14,29–31^. Jiang *et al*. examined the expression of CK7 and CK20 in 26 primary and metastatic BC and found that all the samples were positive to CK7 while 46% showed positive reactivity to CK20^32^. In other studies, the expression of CK20 in urothelial carcinomas was 29%^33^, 83%^34^, 89%,^35^, and 97%^28^. Interestingly, the urine RT-PCR assay of CK20 showed higher sensitivity and specificity for urothelial carcinoma^36^. UPK3A could be a highly specific marker for urothelial tumors in human primary and metastatic urothelial carcinomas^37,38^ as well as dog urothelial tumors^29^. Ramos-Vara *et al*. confirmed the expression of CK7, CK20, and UPK3A in normal dog urinary bladder and 72 dog BC and found that UPK3A is a highly specific and sensitive marker for dog BC, detecting 91% of the examined cases^29^. In our previous study, these three markers were clearly expressed in several BC 3D organoids of dogs and their xenograft-derived tumor tissues^14^. In the present study, they were expressed in 2.5D organoid cells (Fig. 2A) and xenografted tumor tissues (Fig. 4C), indicating that our established method could maintain the urothelial cellular components of their parental 3D organoids and that 2.5D organoid cells mainly consist of urothelial carcinoma cells.

The aggressiveness and prognosis of tumors mainly depend on the population of cancer stem cells (CSCs), the self-renewing and differentiating cells that mediate resistance to chemotherapy^39–45^. CSCs are heterogeneous and composed of several phenotypes with different markers, which renders it extremely difficult to target in an individual patient. Therefore, identifying CSC-specific markers in each tumor is required for the establishment of tailored therapies^46^. Several CSC markers such as CD44, CD133, ALDH1, SOX2, EZH1, PD-L1, CD67LR, BCMab1, BMI1, MAGE-A3, and YAP1^44,47–49^ were known to be expressed in BC. The transcription factor, SOX2 was found to be a CSC marker for both human and mouse BC and was absent in normal urothelial cells^50^. Additionally, SOX2 is associated with tumor progression and prognosis in urothelial carcinoma^51,52^. On the other hand, Chan *et al*. showed that 40% of more than 300 BC samples contained CD44^+^ cells and were able to form tumors *in vivo* 10–200 times than CD44^-^ cells in mice^53^. On the contrary, the CD44^+^/CD24^–^ cells were associated with a better prognosis, indicating that this phenotype is not suitable for detecting CSCs in dog mammary carcinomas^54^. Nevertheless, there is no data showing the expression of CD44 or SOX2 mediates the tumor progression in dog urothelial carcinoma. In the present study, SOX2 was upregulated while CD44 was downregulated in dog BC 3D and 2.5D organoids compared with the 2D urothelial carcinoma cell lines (Fig. 3), suggesting that SOX2 is implicated in the progression and proliferation of BC in dogs by increasing the stemness and could be considered as a novel and reliable CSC marker in dog urothelial carcinoma.

The difference in the drug sensitivities between 2D and 3D culture conditions has been reported in different cancers^55,56^, probably due to higher stemness and expression of multidrug resistance (MDR) proteins in the 3D conditions. In breast cancer, Imamura *et al*. assessed the difference in drug response between 2D and 3D culture of several breast cancer cell lines and primary cultured cells from a patient-derived xenograft and the patient’s original tumor tissues. They found higher resistance to paclitaxel and doxorubicin in the 3D cultured cells compared with the 2D cultured ones, probably due to the decreased expression of cleaved-PARP, cleaved-caspase-3, and Ki-67, greater G0-dormant subpopulation, and hypoxic condition^57^. In BC, Myeong *et al*. showed that the effect of rapamycin and Bacillus Calmette-Guérin (BCG) was more exaggerated in the 2D cell culture than that in the 3D cell culture environment^58^. Additionally, RT4 cells, a BC cell line cultured under 3D conditions also showed higher resistance to doxorubicin compared with 2D cultures^59^. On the other hand, our established 2.5D organoids showed similar responses to anti-cancer drug treatment compared with 3D organoids. Since the trend of stem cell marker expressions in 2.5D organoid cells is more close to 3D organoid ones compared with 2D cell lines as shown in Fig. 3, it might cause the similar response to anti-cancer drugs. Nevertheless, further research is necessary for using 2.5D organoid cells instead of 3D organoids.

## Conclusion

We for the first-time generated BC 2.5D organoids from 3D organoids. These 2.5D organoids showed constant passages and higher proliferation speed than their parental 3D organoids. The 2.5D organoids demonstrated the tumorigenesis *in vivo* and maintained the expression pattern of urothelial and bladder CSC markers. Furthermore, the 2.5D organoids showed a similar response to anti-cancer drugs with their parental 3D organoids. These results suggest that our established gel-free 2.5D culture method can be used as a cheaper and less time-consumed research model instead of 3D organoid studies to investigate the mechanisms of BC diseased dogs. It also provides new insights into the development of dog BC therapy.

## Data Availability

The authors declare that all data supporting the findings of this study are available within the article.
